# The Crucial Role of Atg5 in Cortical Neurogenesis During Early Brain Development

**DOI:** 10.1038/srep06010

**Published:** 2014-08-11

**Authors:** Xiaohui Lv, Huihui Jiang, Baoguo Li, Qingli Liang, Shukun Wang, Qianwei Zhao, Jianwei Jiao

**Affiliations:** 1 State Key Laboratory of Reproductive Biology, Institute of Zoology, Chinese Academy of Sciences, Beijing 100101, China; 2 Key Laboratory of Animal Ecology and Conservation Biology, Institute of Zoology, Chinese Academy of Sciences, Beijing 100101, China; 3 These authors contributed equally to this work.

## Abstract

Autophagy plays an important role in the central nervous system. However, it is unknown how autophagy regulates cortical neurogenesis during early brain development. Here, we report that autophagy-related gene 5 (Atg5) expression increased with cortical development and differentiation. The suppression of Atg5 expression by knockdown led to inhibited differentiation and increased proliferation of cortical neural progenitor cells (NPCs). Additionally, Atg5 suppression impaired cortical neuronal cell morphology. We lastly observed that Atg5 was involved in the regulation of the β-Catenin signaling pathway. The β-Catenin phosphorylation level decreased when Atg5 was blocked. Atg5 cooperated with β-Catenin to modulate cortical NPCs differentiation and proliferation. Our results revealed that Atg5 has a crucial role in cortical neurogenesis during early embryonic brain development, which may contribute to the understanding of neurodevelopmental disorders caused by autophagy dysregulation.

The cerebral cortex of the mammalian brain is a complicated neuronal network that is responsible for the processes of awareness, consciousness, attention, and memory. Neurons are derived from neural stem/progenitor cells during cortical development. The production of neurons is a complex developmental process, known as neurogenesis, which plays key roles in mammalian cortex formation. The mammalian cerebral cortex is composed of multiple layers that are produced by the division of multipotent neural progenitor cells (NPCs)^1^. Progenitor cells are capable of self-renewal and neuron production. The primary progenitor cell, known as the radial glia cell, is not only capable of proliferation and neuron production but also is important for radial neuron migration because their fibers extend from the ventricular zone to the outer surface during cortex development^2^. In the developing neocortex, the process of NPCs proliferation, differentiation, and neuronal migration is regulated by conserved complex interactions of multiple genes and their interaction networks.

Autophagy, as an intracellular bulk degradation process, is highly conserved and plays an important role in many organisms in adaptation to abnormal, stressful conditions. Autophagy is also important for normal biological processes, particularly in homeostatic cells, such as neurons. Autophagy has important roles in a range of physiological events, such as development, immune defense, aging, the prevention of cancer, and neurodegeneration^3^,^4^. There are several types of autophagy, including macroautophagy, microautophagy, chaperone-mediated autophagy, and piecemeal microautophagy of the nucleus^5^. Among these subtypes, macroautophagy is considered the main pathway. Many autophagy-related genes are related to macroautophagy processes. Autophagy-related proteins are also required for autophagosome formation and autophagic function. During autophagosome biogenesis, many autophagy-related proteins function in a hierarchical manner^6^. Previous studies have reported that autophagy is essential not only for homeostasis and protein quality control in neurons but also for neuronal plasticity^7^,^8^. Autophagy is dysregulated in several neurodegenerative disorders^9^,^10^,^11^,^12^. Mice deficient in autophagy-related genes accumulate ubiquitin-tagged cargo and spontaneously exhibit signs of neurodegeneration^13^,^14^,^15^,^16^. Atg5, initially reported in yeast, is a protein essential for the early stages of autophagosome formation^17^. Previous studies have reported the important roles of Atg5 in adult brains^14^,^18^; however, the regulatory roles of Atg5 in the developing neocortex remain unclear. In this study, we investigated how Atg5 regulated neurogenesis during the development of the embryonic cortex.

Here, we report that increased Atg5 expression occurred throughout brain development in parallel with cortical NPCs differentiation in mouse embryos. Furthermore, the loss of Atg5 in the cortex specifically resulted in reduced NPCs differentiation, increased neuronal proliferation, and impaired morphology of cortical neurons. Atg5 modulated β-Catenin stability via autophagy. Moreover, Atg5 together with β-Catenin co-regulated cortical NPCs differentiation and proliferation. These results demonstrated for the first time that Atg5 is a novel regulator of cortical development during certain developmental stages of brain-regulated neuronal differentiation and proliferation. Moreover, we show that this regulation occurs via a previously undescribed mechanism.

## Results

### Atg5 expression in the embryonic cerebral cortex

**Figure 1:**
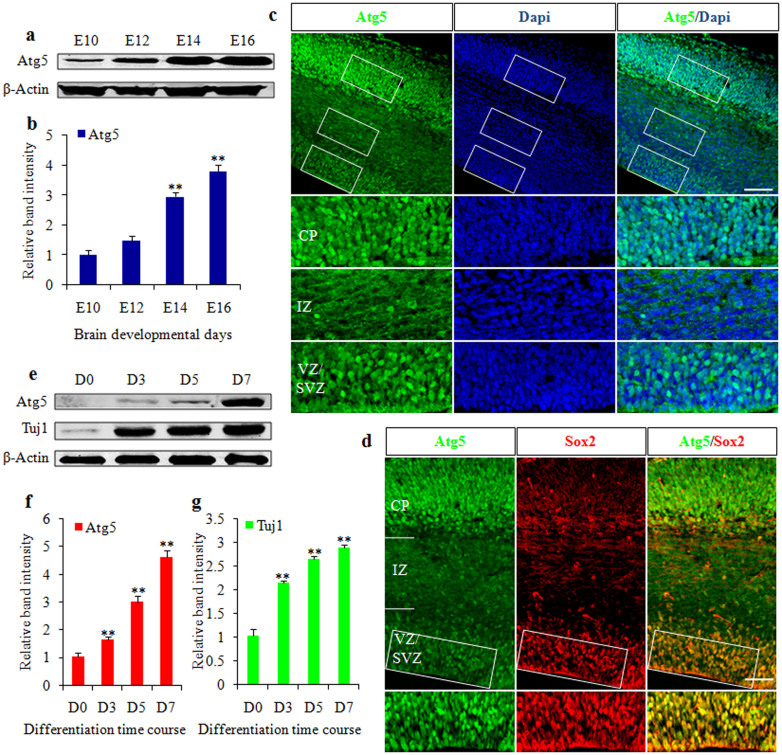
Atg5 expression in the embryonic cerebral cortex. (a). A western blot shows the protein level of Atg5 in the mouse cortex during embryonic development. β-actin was used as a control. Blot images were cropped for comparison. (b). The bar graph shows the relative band intensity of Atg5 from embryonic day 10 to 16 (E10–E16). (c). The expression of Atg5 in the developing cortex. CP, cortical plate; IZ, intermediate zone; SVZ, subventricular zone; VZ, ventricular zone. Boxed areas are enlarged in the bottom. (d). Atg5 is co-localized with Sox2 in the VZ/SVZ of E15 brain sections. Boxed areas are enlarged in the bottom. (e). The protein levels of Atg5 and Tuj1were determined by a western blot in mouse neural stem cells over 7 days of differentiation. β-actin was used as a control. Blot images were cropped for comparison. (f, g).The bar graphs show the relative band intensity of Atg5 (f) and Tuj1 (g) in mouse neural stem cells during a differentiation time course. Scale bars, 50 μm (c and d).

To determine the role of Atg5 in cortical development, we initially examined the expression of Atg5 in the embryonic cortex from embryonic day 10 to embryonic day 16 (E10–E16). A western blot revealed that Atg5 expression increased with embryonic developmental proceeding (Fig. 1a, b). To further investigate the results, we performed the experiments *in vivo*. The immunostaining results displayed higher expression of Atg5 in the cortical plate (CP), subventricular zone (SVZ), and ventricular zone (VZ) in the developing cortex but lower in the intermediate zone (IZ) (Fig. 1c). NPCs mainly reside in the VZ and SVZ^2^, which suggested the possibility of Atg5 expression in NPCs. To examine whether Atg5 was expressed in NPCs, we investigated Atg5 expression in Sox2-positive NPCs using E15 brain sections (Fig. 1d). Double immunostaining revealed that Atg5 was co-localized with Sox2 in the VZ/SVZ, suggesting that Atg5 has important roles in NPCs and may affect neuronal differentiation. To address this possibility, we examined the levels of Atg5 expression with neuronal differentiation. Primary neural stem cells were derived from E12 mouse brains and further cultured over 7 days in the differentiation medium. Notably, a western blot revealed that Atg5 expression increased in parallel with the upregulation of Tuj1 expression during the differentiation time course (Fig. 1e–g). These data suggested the potential roles of Atg5 in cortical NPCs differentiation.

### Atg5 regulates cortical NPCs differentiation and proliferation

**Figure 2:**
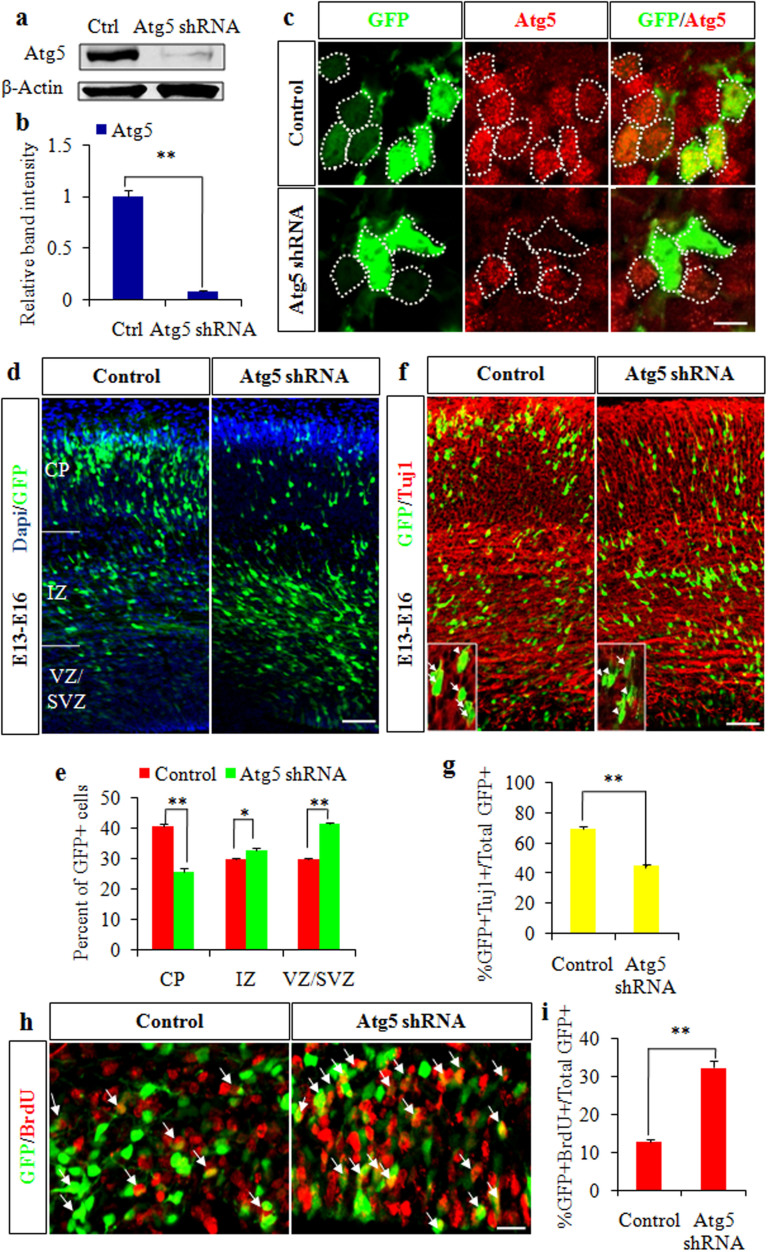
Atg5 regulates cortical NPCs differentiation and proliferation. (a). Control or Atg5 shRNA plasmids were transfected into HEK 293FT cells, and Atg5 protein levels were then analyzed using a western blot. Ctrl, Atg5-expression plasmids plus control shRNA plasmids; Atg5 shRNA, Atg5-expression plasmids plus Atg5 shRNA plasmids. β-actin was used as a control. Blot images were cropped for comparison. (b). The bar graph shows the relative band intensity of Atg5 compared to the control. (c). Atg5 expression is knocked down *in vivo* after Atg5 shRNA plasmid electroporation. Electroporated embryonic brain sections were stained with an Atg5 antibody. The circled cells are GFP-positive cells. (d). Atg5 knockdown changes the cells' positions in the cortex. The control or Atg5 shRNA plasmids were electroporated into the embryonic brains at E13 and harvested at E16. (e). The GFP-positive cell percentages are shown in each region of the cortex. (f). Reduced cortical neuronal differentiation in Atg5 knockdown brains. The E16 brain sections were stained with anti-Tuj1 antibody. Inset shows zoom view of colocalization cells in control and Atg5 shRNA groups. Arrows indicate GFP-Tuj1 double positive cells; Arrowheads indicate GFP+Tuj1− cells. (g). The quantification percentage of GFP-Tuj1 double positive cells relative to total GFP-positive cells is shown. (h). Atg5 knockdown causes increased BrdU incorporation *in vivo*. BrdU (100 mg/kg) was injected into mice 2 hours before sacrifice. The arrows indicate GFP-BrdU double positive cells. (i). The quantification percentage of GFP-BrdU double positive cells relative to total GFP positive cells is shown. Scale bars, 10 μm (c), 50 μm (d and f), and 20 μm (h).

The following experiments were performed to investigate the roles of Atg5. Specific shRNAs that silenced Atg5 expression in cortical NPCs were constructed. We verified the knockdown efficiency of Atg5 shRNA plasmids *in vitro* and *in vivo* (Fig. 2a–c). Given the high expression of Atg5 in cortical NPCs (Fig. 1d) and in the differentiation condition (Fig. 1e–g), we investigated whether Atg5 regulated cortical NPC differentiation. We identified three different small hairpin constructs. E13 embryonic brains were electroporated with control or Atg5 shRNA plasmids and analyzed 3 days later at E16. We observed a significant change of GFP-positive cells in the three cortex zones after Atg5 knockdown. Specifically, we observed an obvious decrease in GFP-positive cells in the CP and a corresponding increase in the SVZ/VZ (Fig. 2d, e). The phenotypes resulting from treatment with the three different shRNA plasmids were identical (data not shown).

The considerable decrease in GFP-positive cell positioning in the CP layer suggested that Atg5 knockdown led to decreased cortical neuronal differentiation. To test this possibility, we stained the electroporated brain with the neuronal marker β-III-tubulin (Tuj1).The quantification results showed a significant decrease in the percentage of GFP-Tuj1 double positive cells caused by Atg5 knockdown (Fig. 2f, g). These results demonstrated that the loss of Atg5 function resulted in a significant reduction in cortical neuronal differentiation. The decreased cortical neuronal differentiation, together with the increased number of GFP-positive cells positioning in the VZ/SVZ layer by Atg5 knockdown, suggested that Atg5 might be involved in enhancing cortical NPCs proliferation. To address this possibility, we investigated whether Atg5 regulated cortical NPCs proliferation. We injected bromodeoxyuridine (BrdU) into mice to label S-phase dividing cells 2 hours before sacrifice. The results showed a marked upregulation in the proportion of GFP-BrdU double positive cells (Fig. 2h, i) following Atg5 knockdown. To preclude the effects of apoptosis on NPCs differentiation and proliferation, we performed a TUNEL assay. The result revealed no significant difference in *in vivo* cell death by the inhibition of Atg5 within electroporated regions compared to the control (data not shown). Together, these results indicated that Atg5 played important roles in cortical cell positioning, differentiation, and proliferation.

### Atg5 knockdown impairs the morphology of cortical neurons

**Figure 3:**
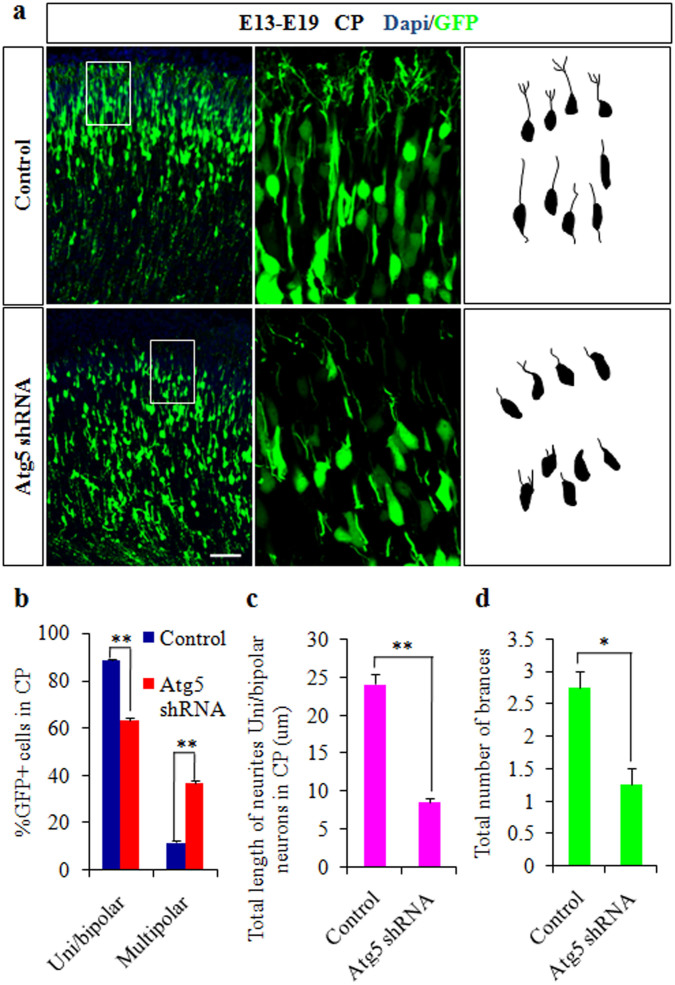
Atg5 knockdown impairs the morphology of cortical neurons. (a). Abnormal morphology of cortical neurons caused by Atg5 knockdown. Control or Atg5 shRNA plasmids were electroporated into E13 brains, and the brain sections were analyzed at E19. (b). The quantification percentage of GFP-positive uni/biopolar or multipolar neurons relative to total GFP positive cells in the cortical plate is shown. (c). The total lengths of uni/biopolar neuron neurites in the cortical plate are shown. (d). The total number of branches of neurons in the cortical plate is shown. Scale bars, 50 μm (a).

Important roles of autophagy in neuronal shaping, connectivity, and development have been reported^10^,^18^. To analyze whether Atg5 knockdown also affects cortical neuronal morphology, E13 brains were electroporated with Atg5 shRNA plasmids and harvested on E19. The neurons located in the CP of Atg5 knockdown did not have the same morphology as control cells (Fig. 3a). A changed ratio of uni/bipolar and multipolar neurons, shorter neurites of uni/bipolar neurons, and lower numbers of branches in the CP were observed after Atg5 knockdown (Fig. 3b–d). These results suggested the involvement of Atg5 in the regulation of axonal and dendritic development. Together, these data support that Atg5 has a crucial role in cortical neuronal morphology.

### Atg5 regulates the β-Catenin signaling pathway

**Figure 4:**
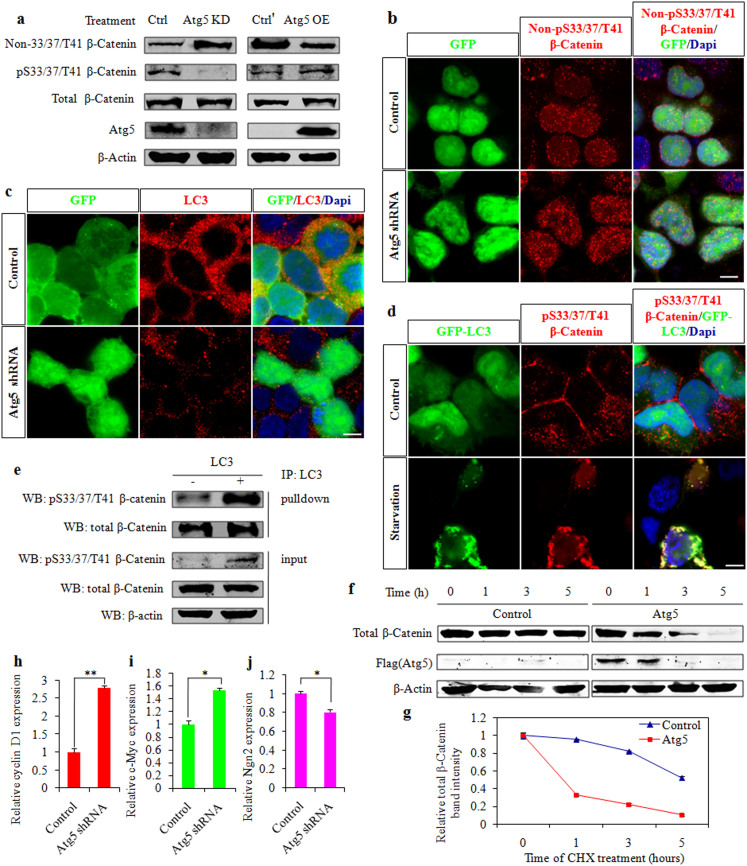
Atg5 regulates the β-Catenin signaling pathway. (a). The effect of Atg5 on the expression of β-Catenin. HEK293FT cells were transfected with Atg5 shRNA, Atg5-expression or control plasmids. Ctrl, Atg5-expression plasmids plus control shRNA plasmids; Atg5 KD, Atg5-expression plasmids plus Atg5 shRNA plasmids; Ctrl', Atg5-expression vector plasmids; Atg5 OE, Atg5-expression plasmids. A western blot was used to analyze the β-Catenin protein levels 3 days later. β-actin was used as a control. Blot images were cropped for comparison. (b). Co-immunostaining shows that the expression of non-pS33/37/T41 β-Catenin increases after Atg5 knockdown. Control or Atg5 shRNA plasmids were transfected into HEK 293FT cells. (c). The expression of LC3 decreases after Atg5 knockdown. Control or Atg5 shRNA plasmids were transfected into HEK 293FT cells. (d). Co-immunostaining shows the expression of pS33/37/T41 β-Catenin and GFP-LC3 in 293FT cells under normal or starvation conditions (in HBSS solution) for 6 hr. (e). LC3 interacts with β-Catenin. Control or LC3 plasmids were transfected into HEK 293FT cells. The transfected cell lysates were subjected to co-immunoprecipitation (co-IP) using an anti-LC3 antibody and immunoblotted with anti-pS33/37/T41 β-Catenin and anti-total β-Catenin antibodies. Input lysates are shown. β-actin was used as a control. Blot images were cropped for comparison. (f). Effects of Atg5 on stability of β-Catenin protein. Flag-Atg5 or empty vector was overexpressed in primary neural stem cells under differentiation conditions. Cycloheximide (CHX, 100 μg/mL) was added after 48 hours. The cells lysates were harvested at the indicated times and immunoblotted with anti-total β-Catenin, anti-flag, and anti-β-actin antibodies. (g). The graph shows the relative band intensity of total β-Catenin relative to the level of β-actin at each time point, compared with the level of β-Catenin at time zero, taken as 1. (h, i, j). Atg5 regulates the downstream target genes (cyclin D1, c-Myc, and neurogenin 2 (Ngn2)) expression of β-Catenin. Primary neural stem cells were infected with control and Atg5 shRNA lentivirus. The cells were harvested after 3 days for real-time PCR. Scale bars, 2 μm (b–d).

The important function of β-Catenin in NPCs proliferation and differentiation during brain development has been reported^19^,^20^,^21^. The regulatory roles of β-Catenin in neurogenesis are developmental stage-specific^22^,^23^. Previous research has demonstrated that β-Catenin overexpression causes the number of cortical precursor expansions, whereas β-Catenin elimination leads to premature neuronal differentiation^24^. Therefore, Atg5 possibly affects neurogenesis through β-Catenin regulation in cortical development. We studied whether the gain or loss of function of Atg5 had similar effects on the expression of β-Catenin using western blotting. We observed that Non-pS33/37/T41 (active) β-Catenin levels decreased when Atg5 was overexpressed, whereas the levels increased with Atg5 knockdown (Fig. 4a). Similar results were reported by previous research^25^. The results suggested that Atg5 might regulate β-Catenin stability. To test this possibility, we performed *in vitro* immunostaining experiments. The results showed an increased expression of Non-pS33/37/T41 β-Catenin after Atg5 knockdown (Fig. 4b). These results revealed the regulatory role of Atg5 in β-Catenin stability. The above results, together with the regulatory roles of autophagy in β-Catenin degradation^26^, indicated that Atg5 activating autophagy might regulate β-Catenin degradation. To investigate this possibility, we initially demonstrated the important role of Atg5 in autophagy. The immunostaining results showed reduced microtubule-associated protein 1 light chain 3 (LC3) expression after Atg5 knockdown (Fig. 4c). Importantly, LC3 serves not only as a marker of autophagosomes but also is important in autophagosome formation^27^,^28^. To further test whether Atg5 was essential for autophagy, we next demonstrated that autophagy levels could not be enhanced by rapamycin when Atg5 was knocked down in primary NPCs (data not shown). We also observed that LC3 gathered and primarily co-localized with pS33/37/T41 β-Catenin under starvation conditions, whereas LC3 was dispersed under normal conditions (Fig. 4d). The co-localization of LC3 with pS33/37/T41 β-Catenin suggested that they may interact. We subsequently tested this possibility using co-immunoprecipitation experiments, observing that pS33/37/T41-catenin associated with LC3 (Fig. 4e). To confirm whether Atg5 affects β-Catenin degradation directly, we followed up the residual level of β-Catenin after cycloheximide (CHX) addition. Data showed that the degradation rate of β-Catenin was accelerated by Atg5 overexpression (Fig. 4f, g). These results indicated that Atg5 regulated β-Catenin stability by activating autophagy. To further study the crucial roles of Atg5 in the β-Catenin signaling pathway, we used a lentivirus system to infect cells and examined changes in the downstream target gene expression of β-Catenin after Atg5 knockdown in NPCs. We observed that the expression of cyclin D1 and c-Myc, downstream target genes of β-Catenin^29^,^30^, significantly increased after Atg5 knockdown (Fig. 4h, i). By contrast, we observed the reduced expression of neurogenin 2 (Ngn2), which is a neuronal determination gene^31^ (Fig. 4j). All of these results indicated that Atg5 regulated the β-Catenin signaling pathway.

### Atg5 cooperates with β-Catenin to mediate cortical NPCs differentiation and proliferation

**Figure 5:**
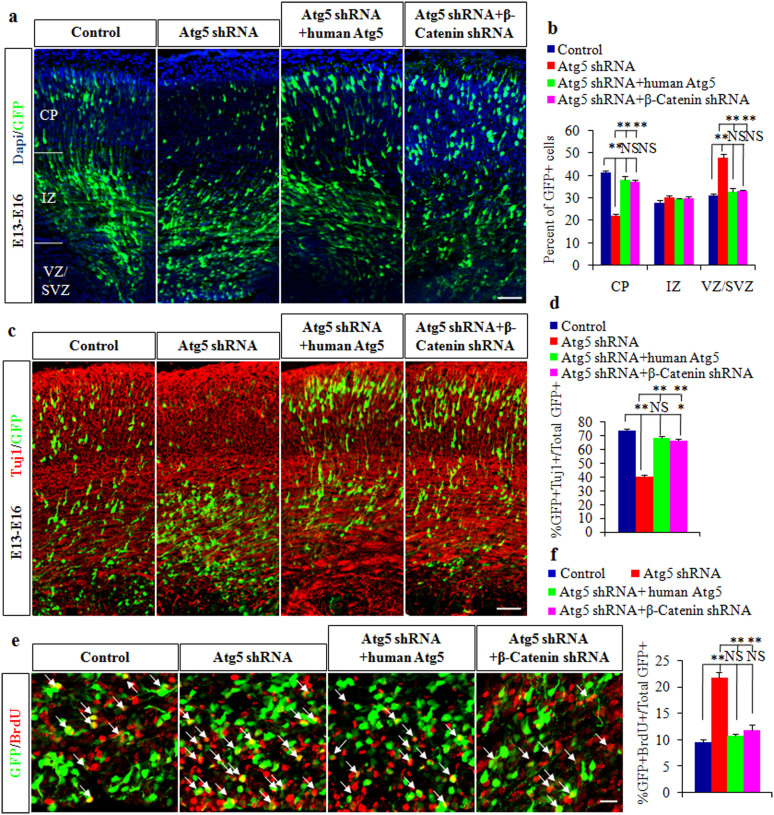
Atg5 cooperates with β-Catenin to mediate cortical NPCs differentiation and proliferation. (a). Defects of cell position caused by Atg5 shRNA are rescued by human Atg5 overexpression or β-Catenin knockdown. (b).The quantification of GFP-positive cells percentage is shown in each region of the cortex. (c). The decreased neuronal differentiation induced by Atg5 shRNA is rescued by human Atg5 overexpression or β-Catenin knockdown. (d).The quantification percentage of GFP-Tuj1 double positive cells to total GFP-positive cells is shown. (e). Increased BrdU incorporation caused by Atg5 knockdown is rescued by human Atg5 overexpression or β-Catenin knockdown in VZ. The arrows indicate double positive cells for GFP and BrdU. (f). The quantification percentage of GFP-BrdU double positive cells to total GFP positive cells is shown. Scale bars, 50 μm (a and c) and 20 μm (e).

Our results demonstrated that Atg5 regulated β-Catenin expression by activating autophagy and suggested that these proteins might coregulate cortical NPCs differentiation and proliferation. We hypothesized that the overexpression of human Atg5 or β-Catenin knockdown could rescue the cellular phenotype caused by Atg5 knockdown in the cortex. To test this possibility, we co-electroporated human Atg5 or β-Catenin shRNA plasmids together with Atg5 shRNA plasmids into E13 brains. Notably, we observed that the Atg5 shRNA-mediated localization defect of GFP-positive cells in the cortex was completely rescued by human Atg5 overexpression or β-Catenin knockdown (Fig. 5a, b). Contrary to the earlier phenotypes caused by Atg5 knockdown, the co-expression of human Atg5 or β-Catenin shRNA with Atg5 shRNA completely rescued these phenotypes. We observed that human Atg5 overexpression or β-Catenin knockdown led to significantly increased neuronal differentiation and reduced BrdU incorporation compared to Atg5 knockdown (Fig. 5c–f). Collectively, these data strongly supported that Atg5 played a key role in regulating cortical NPCs differentiation and proliferation by mediating β-Catenin levels.

## Discussion

In the developing embryonic neocortex, neurogenesis plays a fundamental role in the formation of correct cortex architecture. Neurogenesis relies on internal and external signals and determines the production of the appropriate amount of neurons at the correct time and location. The coordinated roles of multi-genes are crucial for the neurogenesis process during the normal development of the cerebral cortex. Atg5 plays a critical role in the adult brain, and the loss of autophagy causes neurodegeneration^13^,^14^. However, the important role of Atg5 in the developing neocortex remains unclear. It is crucial to know whether or how autophagy controls brain development, as well as whether autophagy loss affects early-stage neuron development. By combining molecular, cellular, electroporation, and anatomical approaches, we report that Atg5 regulates neuronal differentiation, proliferation, and morphology in the developing neocortex. Atg5, as an essential autophagy molecule to maintain basal autophagy, is required for the normal temporal and spatial generation of neurons during brain development. We showed that Atg5 and β-Catenin coregulate cortical NPCs differentiation and proliferation during specific stages of embryonic brain development.

Initially, we observed that the Atg5 levels increased with cortex development. However, the high expression of Atg5 in NPCs suggests that it has potential roles in cortical neurogenesis and brain development. Adult Atg5-deficient mice have defective motor functions and cytoplasmic inclusion body accumulations in neurons^14^. We present several lines of evidence showing that the loss of Atg5 function caused unbalanced cortical NPCs differentiation, proliferation, and positioning. Finely balanced differentiation and proliferation rates in the developing neocortex are a crucial requirement for neurogenesis. There are only a few studies that report the effects of autophagy on NPCs differentiation and proliferation *in vivo*^16^,^32^,^33^. For example, the function of Ambra1 (an autophagy regulator) was demonstrated in Ambra1*^gt/gt^*mice to be important for the balanced of cell proliferation and differentiation^16^. Autophagosomes were nearly absent in neurons of Ambra1-null mice but were present in developmentally normal neurons. In particular, the roles of autophagy-related genes, including Atg5, have been studied in olfactory bulb^32^. However, the mechanism through which autophagy finely regulates cortical neurogenesis during brain development remains unknown. Our results show that autophagosomes are nearly absent in the cortex following the knock-down of Atg5 (data not shown). Previous studies have reported that neurodegeneration is caused by deficient autophagy in the adult nervous system^14^,^15^. In this study, NPCs differentiation and proliferation was unbalanced during cortex development because of autophagy defects, a result that provides additional evidence and explanations regarding the role of autophagy in neurodevelopmental and neurodegenerative disorders. However, we also report that Atg5 overexpression promoted cortical NPCs differentiation and reduced proliferation. Together, these results indicate the importance of Atg5 for NPCs differentiation and proliferation during embryonic cortical development.

In addition, we report that the loss of Atg5 function caused an abnormal morphology of the cortical neurons. The changes in neuronal morphology and connectivity caused by autophagy defects, such as many neuronal abnormalities followed by progressive neurodevelopment, indicate the important role of autophagy in neuronal shaping, connectivity, and development^10^,^18^. Although further investigation is necessary to understand the precise mechanism of neuronal morphology defects, our results provide evidence for neurodevelopmental dysregulation by autophagy loss.

During cortical development, NPCs undergo complex processes, such as proliferation, differentiation, and migration. The balance of proliferation and differentiation in NPCs is critical for the formation of each zone of the cortex. The important roles of β-Catenin in regulating NPCs proliferation and differentiation have been reported^20^,^21^,^22^,^23^,^34^,^35^. In this study, we observed that β-Catenin inhibition increased neuronal differentiation and reduced proliferation in a β-Catenin-knockdown neocortex. β-Catenin was the downstream target of Atg5, and we showed that β-Catenin knockdown could rescue neurogenesis defects caused by Atg5 loss. This result was identical to a previous study that inhibited β-Catenin signaling results in NPC differentiation during embryonic development^24^. β-Catenin is a target for the ubiquitin-proteasome pathway in several cell lines, including neurons, has been proved^36^,^37^. The phospho-β-Catenin-positive aggresomes are formed due to the inhibition of proteasome in rat primary neurons^37^. Previous works have hinted that autophagy has a role in β-Catenin degradation. Rapamycin treatment significantly decreased the level of β-Catenin in Multiple myeloma cells^38^. Degradation of ubiquitin-proteasome pathway substrates could be compromised by autophagy inhibition in HeLa cells and mouse embryonic fibroblast (MEF) cell line^39^. Recent studies have reported that Wnt/β-Catenin represses autophagy and p62 expression, whereas β-Catenin is targeted for autophagic clearance in autolysosomes at autophagy induction^26^. Our study reveals the regulatory role of Atg5 in β-Catenin stability via autophagy. There may be a regulatory feedback mechanism that places β-Catenin at a key cellular integration point, thus coordinating proliferation and differentiation with autophagy.

In summary, our results revealed that Atg5 expression increased with embryonic cortical development and neuronal differentiation. Furthermore, the loss of Atg5 function led to unbalanced cortical NPCs differentiation and proliferation and caused the abnormal morphology of cortical neurons. Moreover, we showed that Atg5 cooperated with β-Catenin to regulate cortical NPCs differentiation and proliferation during brain development. Functionally, human Atg5 overexpression or β-Catenin knockdown fully rescued the deficits in neuronal differentiation and proliferation caused by the loss of Atg5 function. Collectively, these results not only show the expression of and crucial function for Atg5 but also reveal a new mechanism: Atg5-mediated autophagy regulates cortical neurogenesis through the β-Catenin pathway during embryonic brain development. Our results provide additional evidence for, and a better explanation of, the role of basal autophagy during cortical development. Moreover, these results may provide more insight into neurodevelopmental disorders that are characterized by autophagy deficiency.

## Methods

### Animals

Pregnant ICR mice were purchased from Vital River Laboratories. All of the animal studies were performed in accordance with experimental protocols and approved by Animal Care and Use Committees at the Institute of Zoology, Chinese Academy of Sciences.

### Plasmids

Atg5 shRNA and β-Catenin shRNA oligonucleotides were inserted into pSicoR-GFP. The target Atg5 and β-Catenin sequences used were described previously^40^,^41^. The mouse Atg5-expression plasmid was kindly donated from Dr. Wanzhu Jin, and the human Atg5 gene was inserted into pCDH vectors (System Biosciences). Meanwhile, the Flag-tagged mouse Atg5 was cloned into pCMV-Tag 3 vector (Stratagene).

### Cell culture, transfection, and lentivirus infection

HEK 293FT cells were cultured in Dulbecco's modified Eagle's medium (DMEM) supplemented with 10% fetal bovine serum (FBS), non-essential AA, and penicillin/streptomycin in a 37°C, 5% CO2 incubator. The cells were plated and cultured for 24 hours before plasmid transfection. Plasmid transfection was performed using GenEscort I (Nanjing Wisegen Biotechnology) according to the manufacturer's instructions. The lentivirus was packaged with one core vector, psPAX2, and pMD2.G in HEK 293FT cells. Primary neural stem cells were isolated from an E12 mouse embryonic cortex and cultured in the following manner. Briefly, the cerebral cortices were dissected from E12 mouse embryos and dissociated by incubation with Accutase (Life Technologies) for 5 minutes at 37°C followed by washing three times in DMEM containing penicillin/streptomycin. The dissociated cells were cultured in Neural stem cell basal medium (Millipore) supplemented with 10 ng/ml of basic fibroblast growth factor (bFGF) (Life Technologies) and 10 ng/ml of epidermal growth factor (EGF) (Life Technologies) in non-treated Petri dishes as neurospheres to expand the NPCs. The neurospheres were passaged every 3 days. The dissociated cells from the neurospheres in the second passage were replated onto poly-ornithine- and laminin-coated dishes in neural stem cell basal medium supplemented with 5 ng/ml of bFGF and 5 ng/ml of EGF and cultured for 24 hours before infection. To induce differentiation, the medium was replaced with DMEM supplemented with B27 and 1% FBS, and the cells were further cultured for 3–5 days.

### Quantitative real-time polymerase chain reaction (PCR) analysis

The total RNA was extracted from cultured cells using the RNAprep pure Micro Kit (Tiangen Biotech) according to the manufacturer's directions. The complementary DNA was then reverse-transcribed from the total RNA samples using the FastQuant RT Kit (Tiangen Biotech). Quantitative real-time PCR was performed using SuperRealPreMix Plus (SYBR Green I) (Tiangen Biotech) in 20 μl of reaction mixture on an ABI PRISM 7500 sequence detector system (Applied Biosystems). The primer sequences for β-actin, cyclin D1, c-Myc, and neurogenin 2 (Ngn2) were from previous studies^42^,^43^,^44^. The relative amount of each mRNA was determined by the 2^−ΔΔCT^ method^45^. All quantitative real-time PCR studies were repeated three times in triplicate for each sample.

### Western blotting

Protein was isolated from the cultured cells or tissues with lysis buffer RIPA (Beijing Solarbio Science and Technology) supplemented with a protease inhibitor cocktail (Beijing Solarbio Science and Technology). The homogenates were centrifuged at 17,000 *g* for 20 min at 4°C. The supernatants were collected, and the protein concentration was measured using a BCA Kit (Thermo Scientific). Thirty micrograms of protein were loaded into 12% SDS-PAGE gels (Life Technologies) and then transferred to nitrocellulose membranes (Whatman) using the semi-dry transfer system (BIO-RAD). The membranes were blocked with 5% non-fat dry milk (BD Bioscience) dissolved in Phosphate Buffer Saline (PBS) containing 0.05% Tween-20 for 1 hour at room temperature (RT). The membranes were incubated with primary antibodies at 4°C overnight followed by secondary antibodies for 1 hour at RT. The primary antibodies used in the western blot were rabbit anti-LC3 (1:1000, MBL), rabbit anti-Tuj1 (1:2000, Sigma), rabbit anti-non-phospho (Active) β-Catenin (1:1000, Cell Signaling Technology), rabbit anti-phospho-β-Catenin (Ser33/37/Thr41) (1:1000, Cell Signaling Technology), rabbit anti-total β-Catenin (1:800, Proteintech Group), rabbit anti-Flag (1:1000, Sigma), mouse anti-Atg5 (1:500, MBL), and mouse anti-β-actin (1:3000, Proteintech Group). The secondary antibodies were donkey anti-rabbit IgG or donkey anti-mouse IgG (1:10000, Odyssey). The other reagent used in cells for western blotting included cycloheximide (CHX) (Sigma).

### Co-immunoprecipitation

The isolation and concentration measurements of protein from the cultured cells were similar to those used in the western blot. The primary antibody (Ab) was incubated for 1 hour at RT with 50 μl Dynabeads® Protein A (Life Technologies) to form Dynabeads®-Ab complex. After removing the supernatant, the protein samples (50 μg) were incubated for 1 hour with Dynabeads®-Ab complex, and they finally formed Dynabeads®-Ab-Ag complex. After washing three times, 20 μl of elution buffer was added to obtain immunoprecipitation proteins. The supernatant was loaded onto the SDS-PAGE gels, and the relevant binding proteins were assessed by western blotting.

### In utero electroporation

*In utero* electroporation was performed as described previously^46^. Briefly, pregnant dams were deeply anesthetized with Nembutal. Then, 1–1.5 μl of plasmids (1–2 μg/μl) mixed with 0.02% Fast Green (Sigma) were microinjected into the lateral ventricle of the forebrain of E13 mouse embryos. Embryonic brains were electroporated using an electroporator (BTX ECM830) with five 40-V pulses (50 milliseconds duration; 950 milliseconds interval). After electroporation, the brains from the embryos were obtained at different times depending on the experiments and fixed overnight with 4% PFA at 4°C. After dehydration with 30% sucrose for 48 h at 4°C, the brains were embedded in OCT compound (Sakura Finetek) and frozen. The frozen brains were cut coronally at 15-μm thickness. In the rescue experiments, the ratio of the concentration of human Atg5 or β-Catenin shRNA plasmid to Atg5 shRNA plasmid was 2:1. In the proliferation experiments, BrdU (100 mg/kg) was intraperitoneally injected into mice 2 hours before sacrifice, and the brains were harvested for BrdU staining^40^.

### Immunostaining

The brain sections or cell cultures were fixed with 4% PFA for 30 min and blocked in 5% BSA containing 0.1% Triton X-100 (Sigma) for 1 hour. Primary antibody incubation was subsequently performed overnight at 4°C followed by secondary antibody incubation at RT for 2 hours. For BrdU detection, the slices were pretreated with 1 M HCl (10 minutes at 4°C) and 2 M HCl (10 minutes RT and 20 minutes at 37°C) followed by subsequent washes in PBS containing 1% Triton X-100. The brain sections were then incubated with primary antibody overnight at 4°C followed by the secondary antibody for 2 hours at RT.

The antibodies used in the immunohistochemistry and immunocytochemistry steps were mouse Sox2 (1:500, R and D Systems), mouse BrdU (1:1000, Millipore), mouse β-III-tubulin (Tuj1) (1:1000, Millipore), rabbit Tuj1 (1:1000, Sigma), mouse and rabbit LC3 (1:100, MBL), rabbit Atg5 (1:100, Abcam), rabbit β-Catenin (1:400, Cell Signaling Technology), rabbit active β-Catenin (1:400, Cell Signaling Technology), rabbit phospho-β-Catenin (Ser33/37/Thr41) (1:400, Cell Signaling Technology), and rat GFP (1:1000, MBL). The secondary antibodies used were conjugates of Alexa Fluor 488, Cy3, or Cy5 (1:1000, Jackson ImmunoResearch). DAPI (2 μg/μl, Sigma) was used as a nuclear counterstaining.

### Statistical analysis

Statistical analyses, including two-tailed Student's *t*-tests and one-way ANOVAs followed by a Tukey's test for post-hoc multiple comparisons, were performed using SPSS 16.0 for Windows. The values were considered statistically significant (*) at P < 0.05 and highly significant (**) at P < 0.01. The data are presented as the means ± SEM.

## Author Contributions

X.L. and H.J. performed the research, analyzed the data, and wrote the manuscript. B.L., Q.L., S.W., Q.Z. and W.L. performed the research. J.J. designed the research and wrote the manuscript.
